# Genetic signatures of memories

**DOI:** 10.7554/eLife.36064

**Published:** 2018-03-21

**Authors:** Vivek Sagar, Thorsten Kahnt

**Affiliations:** 1 Department of NeurologyNorthwestern University, Feinberg School of MedicineChicagoUnited States; 2 Department of PsychologyNorthwestern University, Weinberg College of Arts and SciencesEvanstonUnited States

**Keywords:** memory, synaptic plasticity, quantitative polymerase chain reaction, immediate-early genes activation, neural activity, expression pattern, Mouse

## Abstract

Memorable positive and negative experiences produce different profiles of gene expression in brain areas associated with long-term memory.

**Related research article** Mukherjee D, Ignatowska-Jankowska BM, Itskovits E, Gonzales BJ, Turm H, Izakson L, Haritan D, Bleistein N, Cohen C, Amit I, Shay T, Grueter B, Zaslaver A, Citri A. 2018. Salient experiences are represented by unique transcriptional signatures in the mouse brain. *eLife* **7**:e31220. doi: 10.7554/eLife.31220

Try to recall your earliest memory. Chances are this experience took place a few decades ago. How did your brain register this event, and then keep a record of it over so many years? These fundamental questions have long been the focus of neuroscience research.

A memory is created when a significant signal is received from the environment and then activates groups of neurons in the brain. Next, genes called ‘immediate-early genes’ are activated in the neurons, and go on to produce a range of proteins that change the strength of the synapses that connect neurons with each other (Sheng and Greenberg, 1990; Kang and Schuman, 1996). It is this synaptic plasticity that drives how memories are created and stored (Martin et al., 2000). Immediate-early gene activation is used as a marker of when and where synaptic plasticity is taking place (Flavell and Greenberg, 2008; Minatohara et al., 2015). Yet, it is not known whether this genetic response includes any specific information about the environmental stimuli that triggered it (Okuno, 2011), or if it is the same regardless of the signal.

Now, in eLife, Ami Citri and colleagues from the Hebrew University of Jerusalem and the Canadian Institute for Advanced Research – including Diptendu Mukherjee, Bogna Marta Ignatowska-Jankowska and Eyal Itskovits as joint first authors – report that different experiences lead to different expression profiles of the immediate-early genes in various regions of brain that are involved in memory in mice (Mukherjee et al., 2018). This makes it possible to infer the type of experience that produced a given expression profile.

First, the mice were given either cocaine (a positive experience) or a substance that made them sick (a negative experience). A method called quantitative polymerase chain reaction (qPCR) was then used to measure the expression of each immediate-early gene in response to the experience. The analysis focused on five brain areas which react to memorable events and play a crucial role in long-term memory (Catani et al., 2013).

Based on these results, Mukherjee et al. selected five immediate-early genes for further analysis. They found that, across the different brain areas, the expression profiles of these five genes were unique for each experience (Figure 1). In fact, the patterns were so specific that it was possible to use them to predict with high accuracy which experience the mouse had gone through. This suggests that the activation profiles for these genes contain information about the specific experiences that an animal encountered.

**Figure 1.**
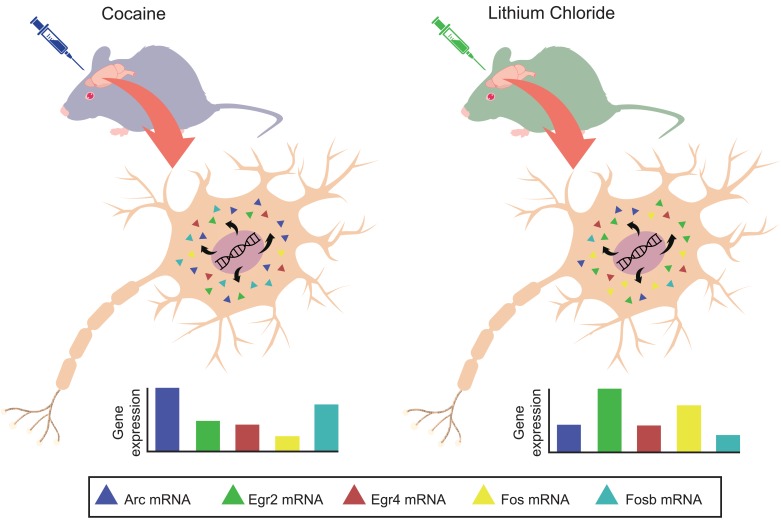
Positive and negative events produce unique gene expression profiles for immediate-early genes in mice. Two groups of mice are exposed to either a positive experience (left) or negative experience (right). This triggers the activation of five immediate-early genes (*Arc*, *Egr2*, *Egr4*, *Fos* and *Fosb*; different color triangles) in the neurons of brain regions that are involved in the formation of long-term memories. The level of expression of each gene is quantified using quantitative polymerase chain reaction (qPCR). The expression pattern of these five genes (bottom) is specific to the event experienced by the mice.

IMAGE CREDIT: Vivek Sagar and Thorsten Kahnt.

To confirm these results, further experiments were conducted in which mice were exposed to additional positive and negative experiences (such as being given sucrose or an electric shock). Again, each event was associated with a characteristic gene activation profile, and the specific experience could be predicted on the basis of gene expression patterns. Strikingly, positive experiences (e.g. cocaine and sucrose) produced gene expression profiles that were similar to each other across the five brain areas; so did the negative events. However, the profiles produced by negative and positive signals were different from each other. Moreover, it was still possible to discriminate between the patterns created by different types of positive (or negative) experiences.

In a final step, Mukherjee et al. used their decoding model to identify the smallest combination of genes and brain areas that was sufficient to predict individual experiences with high accuracy. This minimal subset contained only eight gene-brain area combinations, and was better at predicting experiences than any of the genes or brain regions in isolation.

The results of this study suggest that immediate-early genes are not merely indicators of synaptic plasticity. Rather, the expression profiles for these genes contain fairly specific information about the stimulus that created them. This is similar to what has been observed with patterns of neural activity (Howard et al., 2015). This specificity provides important insights into how to store the identity of an experience.

These experiments provide a solid foundation from which to explore the mechanisms related to the activation of immediate-early genes in greater detail. For example, we do not know whether the same processes will operate in regions of the brain that are not associated with memory. It also remains unclear if highly similar experiences would still produce distinguishable genetic signatures.
